# Equity for women and underrepresented minorities in STEM: Graduate experiences and career plans in chemistry

**DOI:** 10.1073/pnas.2020508118

**Published:** 2021-01-11

**Authors:** Jean Stockard, Celeste M. Rohlfing, Geraldine L. Richmond

**Affiliations:** ^a^Department of Planning, Public Policy and Management, University of Oregon, Eugene, OR 97403;; ^b^Committee on the Advancement of Women Chemists (COACh), University of Oregon, Eugene, OR 97403;; ^c^Department of Chemistry, University of Oregon, Eugene, OR 97403

**Keywords:** graduate student experience, underrepresented minorities, URM, women

## Abstract

Recent events prompted scientists in the United States and throughout the world to consider how systematic racism affects the scientific enterprise. This paper provides evidence of inequities related to race–ethnicity and gender in graduate school experiences and career plans of PhD students in the top 100 ranked departments in one science, technology, engineering, and math (STEM) discipline, chemistry. Mixed-model regression analyses were used to examine factors that might moderate these differences. The results show that graduate students who identified as a member of a racial/ethnic group traditionally underrepresented in chemistry (underrepresented minorities, URM) were significantly less likely than other students to report that their financial support was sufficient to meet their needs. They were also less likely to report having supportive relationships with peers and postdocs. Women, and especially URM women, were significantly less likely to report supportive relationships with advisors. Despite their more negative experiences in graduate school, students who identified as URM expressed greater commitment to finishing their degree and staying in the field. When there was at least one faculty member within their departments who also identified as URM they were also more likely than other students to aspire to a university professorship with an emphasis on research. Men were significantly more likely than women to express strong commitment to finishing the PhD and remaining in chemistry, but this difference was stronger in top-ranked departments. Men were also more likely than women to aspire to a professorship with an emphasis on research, and this difference remained when individual and departmental-level variables were controlled.

## Significance

On June 10, 2020, as part of the Black Lives Matter movement, scientists in the United States and throughout the world paused to consider how systematic racism affects the scientific enterprise. As a result, many academic departments are now assessing policies and practices that may contribute to this situation. This paper provides evidence of the nature of inequities related to race–ethnicity and gender in graduate school experiences and career plans of PhD students in one science, technology, engineering, and math (STEM) discipline, chemistry. The results can help promote understanding of the problems and guide efforts toward equity within STEM and, potentially, other academic areas. In turn, these changes can strengthen the scientific enterprise and the well-being of society.

Fifty years ago, the field of chemistry was overwhelmingly populated by white men, but, in recent decades, has become more diverse. Since 2000, women have received about half of the bachelor’s degrees and over a third of the PhDs. The representation of those who identify as underrepresented minorities (URM), including African Americans, Latinx, and Native Americans, has also increased, reaching 22% of bachelor’s and 12% of PhD degrees awarded in 2016 (1). These percentages, however, are far less than their representation within the US population (∼30%). Moreover, representation in faculties at research-intensive colleges and universities has remained lower than would be expected given the number of PhD recipients. By 2016, only a little more than one-fourth of tenure-track professors at the assistant and associate ranks in top-ranked departments were women. Substantially fewer (6%) identified as URM (2–6).

Research has highlighted the importance of the educational process in increasing the representation of women and URM in science, technology, engineering, and math (STEM) (7, 8), but less research has focused on the nature of graduate school experiences and their relationship to career plans within a specific STEM discipline (9). In this paper, we begin to fill this gap by looking at the extent to which graduate school experiences and career plans of chemistry graduate students differ by gender or identification as URM, and factors that might moderate, or help explain, these differences. Data came from a 2013 survey of chemistry graduate students sponsored by the American Chemical Society (ACS) (10) and publicly available data on chemistry departments. The sample was restricted to doctoral students enrolled in the 100 departments in the United States that receive the largest share of research funding and who had been enrolled in their departments from 1 to 5 y. It included 1,375 graduate students, with an average of 13.8 students in each department (range 1–53).

We found disturbing patterns of inequitable graduate experiences and career plans. On average, women, and especially those who identified as URM, reported significantly fewer positive interactions with their advisors than other students. URM students were less likely to report that financial support for their graduate studies was adequate to meet their needs. This difference in financial support was slightly smaller, but remained, in even the most prestigious and resource-rich departments. URM students, and especially men, were significantly less likely to report receiving interpersonal support that they desired. Women were significantly less likely than men to be committed to finishing their PhD and staying in chemistry, but this pattern was strongest among students in larger or more prestigious departments. Women were also significantly less likely to aspire to professorships with an emphasis on research, rather than teaching, and this association was not moderated by any of the control variables we introduced. Although the results are concerning to US chemistry departments, the findings have relevance to graduate student equity issues across the STEM disciplines and other areas of academe.

## Background

A relatively large literature has examined variables associated with students’ persistence in STEM degree programs. Factors often cited as important include strong relationships with an academic advisor or mentor (7, 11), support from peers and coworkers, and a “sense of belonging” or “community” (7, 11–19). Researchers have documented the way in which subtle negative interactions, incivilities, or “microaggressions” can negatively impact student experiences and plans (20–24), and have stressed the importance of promoting true inclusion rather than simple numerical diversity (25). Based on this literature, we examined students’ perceptions of their relationships with advisors and peers.

There are also documented differences in student experiences and outcomes between departments and universities with varying levels of prestige and different faculty composition. Both women and students identifying as URM appear less likely to finish STEM degrees in more prestigious schools (3, 15, 26). For students who identify as URM, the presence of faculty who also have this identification appears important. A study of chemistry doctoral programs found that departments with more faculty members identified as URM had a greater increase over time in the percentage of URM students receiving PhDs. However, this study also found a slight negative association between the representation of women faculty and PhDs awarded to women (3). Previous studies have also reported lower levels of financial support for URM students (11, 27). Based on this literature, we examined variations in students’ experiences across departments with different characteristics as well as differences in financial support related to gender or identification as URM. Note that these studies, as well as our own, underrepresent PhD programs in historically Black colleges and universities (HBCUs) as most fall outside of the top 100 institutions as ranked by research funding.

Individual characteristics other than gender and identification as URM can, of course, influence graduate experiences and career plans. Researchers have noted the way in which retention in graduate programs is enhanced by support from significant others, especially family members (11), and higher socioeconomic status (28, 29). In addition, individual values and preferences have been found to influence aspirations (30). It is possible that women chemists have more often opted for nonacademic careers because they prefer work environments that allow greater freedom to pursue family and other personal interests. Thus, we included measures of these variables in our analysis to see if any differences related to gender or identification as URM might be moderated, or lessened, when these variables were considered.

## Materials and Methods

### The Study of the ACS Data Was Reviewed and Deemed Exempt by the University of Oregon Human Subjects Office.

The ACS summary of the survey responses (10) noted significant bivariate results related to gender and identification as URM but did not attempt to examine why differences appeared or test hypotheses regarding the association of graduate experiences with factors related to the students and their departments. Our work was designed to fill this gap and was based on the assumption that effective actions to address inequities require understanding the underlying dynamics.

Our analysis of the data obtained from the ACS focused on three measures of the graduate school experience: quality of relationship with the advisor, support received from other graduate students and postdocs, and perceived adequacy of financial support; and two measures of career-related plans: commitment to finishing the PhD degree and staying in chemistry, and intent to pursue a career as a professor in a research-oriented university. To test for moderating effects, we examined the impact of six individual-level variables (years in the program, parental education, marital/partnered status, having dependents, value attached to having a well-paid and secure career, and value attached to having a job with flexibility for family and personal interests) and four department-level variables (a composite measure of the size and prestige of the department, diversity of the university, proportion of women on the faculty, and any department faculty who identified as URM).

We used mixed-model regression analyses, a powerful statistical method for examining associations among variables measured on two levels of analysis (individual students and their departments). We examined a series of increasingly more complex models, looking at 1) the extent to which experiences and plans differed by gender and identification as URM, and 2) if these differences were moderated by the individual and department-level control variables. The analysis included tests of interaction effects, or the ways in which the “intersection” (31) of gender and URM might result in different experiences. The final, best-fitting models were determined through standard model fit procedures. (*SI Appendix* gives details on the underlying theoretical model, methodology, and results.)

## Results

Slightly more than half (52%) of the graduate students who responded to the ACS survey were men. Nine percent identified as URM, and this percentage was similar for men and women. On average, students had been in their programs for about two and a half years. Slightly more than one-fourth were first-generation college students, but this percentage was significantly higher for students who identified as URM. About two-fifths were married or partnered but less than one-tenth indicated that they had dependents. On average, students indicated that obtaining a secure and well-paid job was relatively important (4.0 on the 5-point scale), and students who identified as URM were significantly more likely to express this view. Students were slightly less likely to attach value to family-related and other personal aspects of their future jobs (3.7 on the 5-point scale). Women and those who identified as URM were more likely to attach greater value to these elements. There were no gender differences in the contextual measures. That is, women and men were, on average, in schools of similar rank, similar levels of diversity, and with similar proportions of women and URM faculty. Students who did or did not identify as URM were in schools with similar levels of campus and faculty diversity. But, those who identified as URM were significantly less likely to be in larger and more prestigious departments. In total, 38% of the students were in schools within the top quartile of our measure of departmental size and prestige compared to 30% in departments in the bottom half of this measure. (Details in *SI Appendix*, Table S1.)

### Graduate School Experiences.

**Fig. 1.**
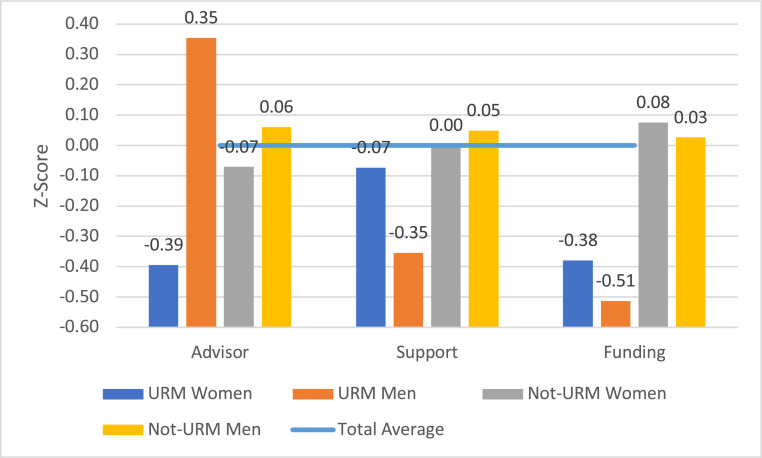
Average standardized (z) scores, measures of graduate experiences by gender and identification as URM. Note: The sample included 63 women identified as URM, 64 men identified as URM, 590 women not identified as URM, and 658 men not identified as URM.

Fig. 1 shows average values on the measures of graduate school experiences for men and women who did or did not identify as URM. Because the measures had slightly different scales, we transformed the values to standardized (z) scores. By definition, standardized scores have a mean of zero and an SD of 1, and thus the values for each variable are comparable. The horizontal line in the graph represents the overall average (zero) for the total group. When a bar associated with a group falls below that line it indicates experiences that were less favorable than the overall average. The average values for each group can also be compared, yielding the difference, in SD units, between average scores for students in two groups. (Details in *SI Appendix*, Table S2.)

#### Advisor–student relationships.

The survey asked an extensive series of 22 questions about students’ experiences with their advisors in areas such as involvement in research, availability, encouragement, and fair treatment. Because responses to these measures were highly correlated, we combined them into an additive scale (Cronbach’s alpha = 0.94). Some students (9% of the total) reported that they had two advisors and the scale score for these students was the average of the ratings given to the two advisors.

Generally, students reported that their advisors were slightly supportive (an average of 3.6 on the 5-point scale). However, men were more likely than women to say that their advisors were supportive, and this gender difference was greater among those identifying as URM (a significant interaction effect). Women identifying as URM reported the most negative experiences (an average z score in Fig. 1 of −0.38), while men identifying as URM reported, on average, the most positive (an average z score in Fig. 1 of +0.23). We found similar differences in each of the 22 individual items in the scale. Women identifying as URM were least likely to report that their advisors encouraged them to take challenges or pursue their goals, advocated for them, gave credit for their contributions, created a “fair environment,” gave regular feedback, engaged them in writing proposals and giving presentations, helped develop professional relationships, or indicated that they were satisfied with the student’s work.

Our multivariate statistical analyses indicated that the more negative experiences of URM women could not be explained by the other variables we examined. In other words, no matter what year the students were in their graduate program, the educational level of their parents, their marital status, the value attached to different aspects of their careers, the size and prestige of their department, the diversity of their university, or the composition of their faculty, the URM women were significantly more likely than other students to report negative experiences with their advisors. Women who did not identify as URM had the next most negative experiences (details in *SI Appendix*, Tables S3–S5).

#### Support from peers and postdocs.

A series of survey questions asked the students about the extent to which they desired and received “support and advice” regarding their “professional development and career” from others. We focused on support from other graduate students and postdocs, the two groups with which they would be most likely to interact on a day-to-day basis, and combined these indicators into a 3-point scale with the highest value indicating that students received as much support as they wanted from both sources and the lowest indicating that they did not receive this level of support from either source.

On average, non-URM men were most likely to report that they received the support they desired, followed by non-URM women. Students who identified as URM, and especially URM men, were less likely to report receiving such support. (The interaction effect was statistically significant.) Almost a quarter of URM men (24%), compared to 16% of the URM women and 12–13% of the non-URM students, reported that they did not receive desired support from either fellow graduate students or postdocs. Less than half of the URM men, but almost two-thirds of the non-URM men, reported that they received the support they wanted from both sources. Our multivariate analyses indicated that this pattern was not moderated by any of the individual or department-level factors. (Details in *SI Appendix*, Tables S3–S6.)

#### Financial support.

In addition to questions regarding advisor relations and support from others, students were asked to indicate the extent to which they agreed or disagreed with the following statement: “The funding for my graduate studies is adequate to meet the cost of living where I live.” Responses could range on a 5-point scale from strongly disagree to strongly agree. On average, there were no differences between men and women in answers to this query. However, there were significant differences between students who identified as URM and those who did not. Students who identified as URM were more than twice as likely as other students to indicate that their financial support was not adequate (35% versus 16% responding disagree or strongly disagree). The z score associated with URM men was especially low: −0.51 or greater than one-half of an SD below the overall mean. The students were also asked to report what percentage of their support came from sources such as teaching assistantships (TA), research assistantships (RA), fellowships, and personal resources including other employment, support from family members, and loans. Reflecting the inadequacy of formal graduate stipends, the percentage of support from personal resources was over twice as large for URM students as for other students (7.1% versus 3.2%, t = 3.86, *P* < 0.001).

**Fig. 2.**
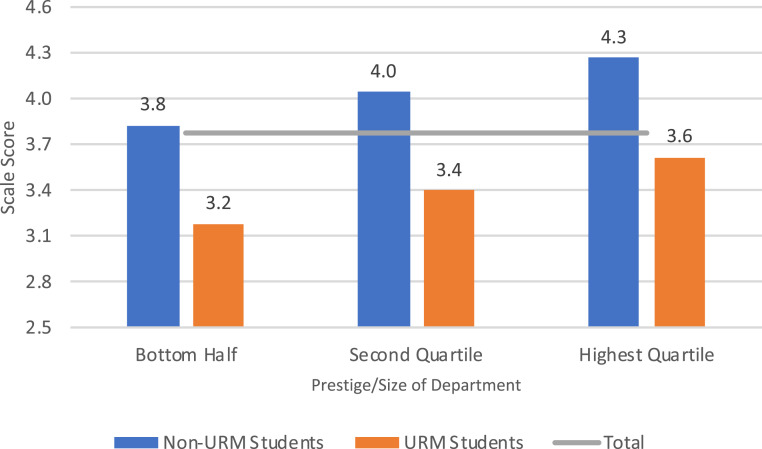
Estimated average values, perceived adequacy of financial support by prestige/size of department and identification as URM, controlling for other individual and departmental variables. Note: Perceived adequacy of support measured on a 5-point scale with 5 indicating strongly agree. Mean values estimated from the best-fitting mixed-model regression and included controls for gender, marital status, dependents, value attached to a high paying job, diversity of university, and presence of URM faculty in the department. Estimated means for the measure of departmental prestige and size calculated at the midpoint of each range shown. Horizontal line indicates the average value for all students. Thirty percent of the students (361 non-URM and 47 URM) were in the 50 departments in the bottom half of the prestige/size distribution of departments; 33% (401 non-URM and 44 URM) in the 25 departments in the second quartile; and 38% (486 non-URM and 36 URM) in the 25 departments in the highest quartile. Additional details in *SI Appendix*.

The results of our multivariate analyses indicated that the difference between URM and other students in perceived adequacy of support was not moderated by students’ individual characteristics, such as marital status or having dependents; the extent to which they received support from TAs, RAs or fellowships; nor by the composition of departmental faculty or diversity of their universities. It was, however, moderated by departmental prestige and size. The gap in reported adequacy of funding between URM and other students was less in departments that were larger and had more research funding, but did not disappear. Fig. 2 illustrates these results by displaying the average value on this measure, predicted by our best-fitting statistical model, for URM and non-URM students in schools at various levels of size and prestige. The values in the figure show the average for each group that would be predicted if they were equivalent on each of the variables included in the analysis, including both individual and departmental characteristics. (Details in *SI Appendix*, Tables S3, S4, and S7–S9.)

Taken together, our findings regarding graduate school experiences support earlier research and theoretical understandings. Women’s less satisfactory relationships with advisors could be due to gender-biased assumptions regarding women’s roles in STEM. The inequities encountered by URMs could be due to institutional racism. The lack of personal support from peers illustrates the ways in which implicit biases and a lack of true inclusion can permeate social interactions in daily life (20–25). The greater difficulties with funding can be linked to an almost 10-fold difference in net worth between non-Hispanic whites and minority groups that are underrepresented within STEM (*SI Appendix*, Table S14). Even though departments and funding agencies generally have well-established and seemingly universalistic student funding policies, URM students could be far less likely to have back-up financial resources and be more likely to face expectations from others, such as family members, for financial assistance. While our analysis controlled for family background and having dependents, it is unlikely that these control variables were adequate to fully capture the true scope of financial disparities. At present, graduate students are paid at levels far less than they might earn outside academe and the impact of this low level of funding appears to be far greater for URM students than for others.

### Career Plans.

**Fig. 3.**
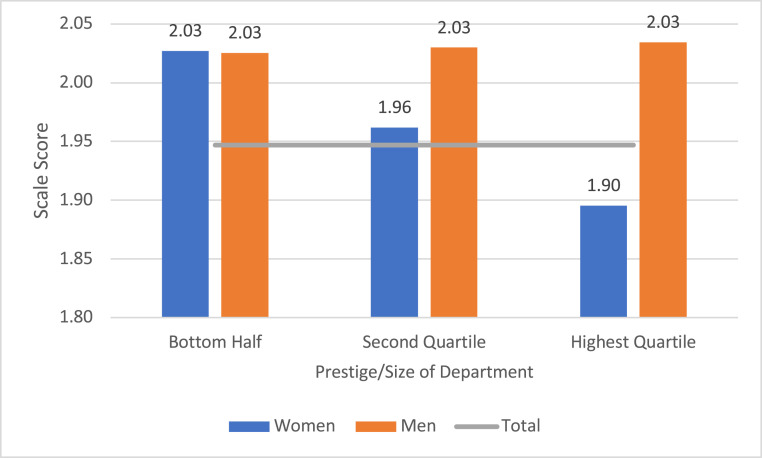
Estimated average values, commitment to finishing degree and staying in chemistry by prestige/size of department and gender, controlling for other individual and departmental variables. Note: Commitment was measured on a 3-point scale, with 3 indicating strongest commitment. Mean values estimated from results of the best-fitting mixed-model regression and included controls for identification as URM, years in the graduate program, marital status, value attached to a high-paying job, value attached to a job allowing time for family and other interests, quality of advisor, and presence of URM faculty in the department. Within all departments both men and women students reported greater commitment when they also reported more supportive advisors. Horizontal line is the average scale score for all students. Thirty percent of the students (183 women and 225 men) were in the 50 departments in the bottom half of the prestige/size distribution of departments; 32% (206 women and 239 men) in the 25 departments in the second quartile; and 38% (264 women and 258 men) in the 25 departments in the highest quartile. Additional details in *SI Appendix*.

In the second part of our analysis we examined students’ career-related plans and the extent to which any differences related to gender and identification as URM were moderated by characteristics of the students, their departments, and their graduate experiences. We first looked at students’ commitment to finishing the PhD and remaining within the chemical sciences. Men were significantly more likely than women to express high commitment; within each gender group, those who identified as URM were more likely to do so. (The standard scores, or z scores, associated with this measure were 0.25 for URM men, 0.07 for non-URM men, 0.003 for URM women, and −0.11 for non-URM women.) Our multivariate analyses indicated that gender differences, but not those related to URM status, were moderated by other variables, specifically the size and prestige of the department and the quality of the relationship that students had with their advisors. Students who had more supportive advisors expressed significantly greater commitment to the field and their degree, and this association was similar for men and women. In contrast, the association between the prestige of the department and commitment varied for men and women, as illustrated in Fig. 3. There was no gender difference in commitment in the less prestigious and smaller departments, but striking differences in the more prestigious schools, where women were significantly less likely to be committed to completing the PhD and remaining in chemistry. (Again, the values depicted in Fig. 3 control for, or equalize, other variables related to commitment. Additional details in *SI Appendix*, Tables S10–S12.)

The second measure of career plans involved students’ career aspirations, focusing on the area in which women have been most underrepresented––tenure-track professorships at research universities. The highest value of this measure indicated students were very interested in completing a postdoc and becoming a professor with an emphasis on research, and the lowest value indicated no plans for either pursuit. [The phrasing of the question differentiated “professor (emphasis on research)” from “professor (emphasis on teaching)” as well as “researcher (not professor) in college/university.”] The pattern of differences by gender and identification as URM paralleled those with the measure of commitment. Men were significantly more likely than women to aspire to a postdoc and professorship; and, within each gender group, those who identified as URM were more likely than other students to express these aspirations. (The standard scores, or z scores, associated with this measure were 0.52 for URM men, 0.19 for non-URM men, −0.19 for URM women, and −0.25 for non-URM women.)

**Fig. 4.**
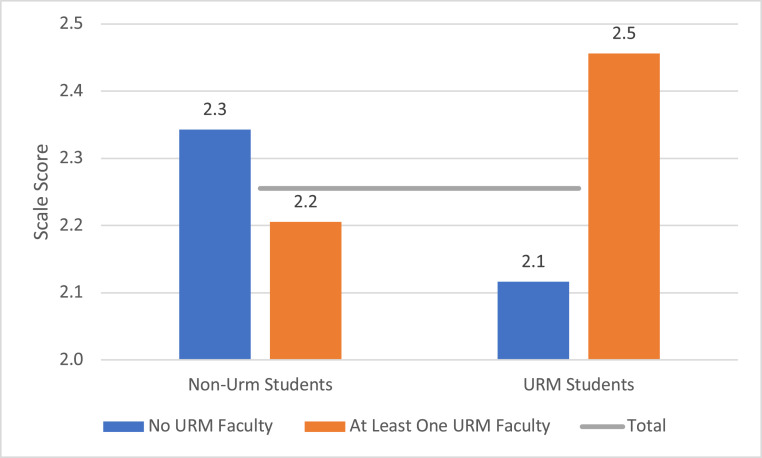
Estimated average values, career aspirations by identification as URM and presence of URM on faculty, controlling for other individual and departmental variables. Note: Aspirations for a faculty position at a research-oriented institution measured on a 4-point scale with the highest value indicating greatest commitment. Estimates derived from results of mixed model regression that included controls for gender, parental education, value placed on a job allowing time for personal interests and family, supportive relations with advisor, support from peers and postdocs, and commitment to finishing degree and staying in the chemical sciences. The horizontal line is the average for the total group. Among the URM students, 81% were in departments with at least one URM faculty. Among the non-URM students, 74% were in such departments. Seventy of the 100 departments had at least one URM faculty member. Additional details in *SI Appendix*.

Multivariate analyses indicated that students were significantly more likely to aspire to a professorship emphasizing research when they were first-generation college students, attached less importance to a job that allowed time for family and other interests, had a more supportive advisor, and expressed greater commitment to finishing their degree and staying in chemistry. Yet, even when all of these variables were equalized, women were less likely than men to express this aspiration. At the same time, however, the multivariate results revealed that the tendency for students who identified as URM to more often plan to become a professor at a research-oriented university was highly dependent on the composition of their departmental faculty. As illustrated in Fig. 4, in departments with at least one faculty member identified as URM, the URM students were more likely than others to express strong aspirations to a postdoc and professorship in a research-oriented department. In contrast, URM students in departments without any professors identified as URM were slightly less likely than other students to express these aspirations. The difference between non-URM students in the two types of departments was much smaller. (Details in *SI Appendix*, Tables S10–S12.) (As noted above, our study of the ACS survey data underrepresents PhD chemistry programs in HBCUs as most fall outside of the top 100 institutions as ranked by research funding.)

Our analysis of students’ career plans illustrates the important role of both a supportive advisor and departmental-related variables in students’ career plans, supporting previous literature reviewed above. Net of other variables, students who had more supportive advisors were more likely to plan to finish their degree, stay in chemistry, and aspire to a postdoctoral position and an academic career at a research-oriented university. Yet the positive effect of an advisor on women’s commitment was muted in larger and more prestigious departments, where the gender gap in commitment was significantly stronger. The racial/ethnic composition of departments’ faculties was especially important in explaining the aspirations of URM students, paralleling earlier research on chemistry departments (3). We suggest that these results highlight the ways in which those concerned with graduate students’ career plans need to address both the actions of individual faculty as well as the nature of departmental culture and composition.

## Conclusion

For many years, members of the scientific community have stressed the importance of diversity and inclusion within the scientific enterprise by finding and building on scientific interests and talents from all segments of the population (23, 32, 33). Graduate schools are the major path to producing a diverse and inclusive scientific workforce. Yet, as illustrated in this paper, graduate school experiences can mirror inequities in other areas of the society and potentially work against achieving this goal. The academic world projects an aura of universality, meritocracy, and respect for all, regardless of their individual characteristics. However, the results of this analysis suggest that the reality for those who are traditionally underrepresented appears to be quite different. In their academic departments and laboratories, these underrepresented students may encounter subtle, insidious, and continual social and psychological hostilities and devaluation. Amazingly, despite this situation, students who identify as URM are more likely to plan to persist in their degree programs and the discipline, and to aspire to careers as professors who emphasize research as well as teaching. This commitment suggests extraordinary individual courage and devotion to their science.

The ACS is commended for commissioning the data-gathering effort that led to this paper. It could be tempting for some to dismiss the findings described above as unique to one discipline, or to STEM but not to other areas. Given the deep roots of systematic racism and sexism within our society, it would seem unlikely that the results reported here are so limited. At the very least, this is an empirical question and one that requires concerted attention throughout the scientific and academic world. Thus, we hope that researchers replicate this work in a wider range of institutions, including HBCUs and those with less research funding, in other STEM fields, and in other academic disciplines. *SI Appendix* includes an extended discussion of possible future research as well as the implications of our findings for changing policies and practices. However, it is important to note that ample resources are available to assist chemistry departments and faculty who wish to address these findings, including minority technical organizations such as the National Organization for the Professional Advancement of Black Chemists and Chemical Engineers (34) and the Society for Advancement of Chicanos/Hispanics and Native Americans in Science (35).

In June 2020, many scientists in the United States and throughout the world expressed their commitment to uncovering and addressing inequities within their disciplines and to demonstrating that Black and brown lives matter within laboratories, hallways, faculty offices, and the everyday life of academe and the larger scientific enterprise (36). We hope that the findings summarized in this paper can help guide corrective actions, not just within chemistry, but throughout academia.

## Supplementary Material

Supplementary File

## Data Availability

Data is available at https://coach.uoregon.edu/coach-research-publications-and-articles.
