# Supplementary material for: Equity for women and underrepresented minorities in STEM: Graduate experiences and career plans in chemistry
Source: Proc Natl Acad Sci U S A. 2021 Jan 11;118(4):e2020508118. doi: 10.1073/pnas.2020508118 (PMC7848687; doi:10.1073/pnas.2020508118)
Supplement: Supplementary File [file pnas.2020508118.sapp.pdf]

Supplemental Information
Equity for Women and Underrepresented Minorities in STEM:
Graduate Experiences and Career Plans in Chemistry
Supplemental Information
November 4, 2020

This document provides details beyond those presented in the text of the article. The sections that follow discuss methodology, results, and implications for future research and policy.

Methodology

Sample

As noted in the text, the sample was limited to PhD students in chemistry departments ranked in the "top 100" in terms of research funding as reported by the National Science Foundation.[1] We focused on these departments because they train the majority of graduate students and because reliable data on two important contextual measures (the presence of women and people who identified as URM on the departmental faculty) were not available for the unranked departments. Students who identified as URM were more often in the unranked departments (17% versus 10%) as were those who were first generation college students (16% versus 8%). However, analyses not included within this paper indicated that substantively similar results appeared when students in the "non-ranked" departments were included. Results with this sample are available upon request from the corresponding author.

The sample was further restricted to those who had been enrolled in their departments from one to five years. This ensured that students would have enough experience within their program to provide more valid information and also omitted those who, for whatever reason, had remained in their program substantially longer than other students. Seventeen percent of the students who responded to the survey were new to their graduate program and omitted for that reason. Only five percent (n=95) had been in their program for 6 years or more, and

[1] https://ncsesdata.nsf.gov/herd/2016/html/HERD2016_DST_49.html

over half of these students (3.4% of the total) had been in their program for 12 or more years. However students who identified as URM were slightly more likely to be in these groups.

Students were also omitted if they did not provide data on gender, racial-ethnic identification, or the dependent variables. The final sample included 1,375 graduate students in 100 departments. There were an average of 13.9 students in each department, with a range of 1 to 53. A total of 127 students identified as URM. Close to half of these students (45%) identified as Latinx. They were equally divided between men and women.

Measures

Dependent Variables. Three measures were developed to indicate students' graduate school experiences. An extensive series of 22 questions asked about students' experiences with their advisors, involving areas such as involvement in research, availability, encouragement, and fair treatment. Because responses to these measures were highly correlated they were combined into an additive scale (alpha = .94). Some students (9% of the total) reported that they had two advisors and the scale score for these students was the average of the ratings given to the two advisors. Another series of questions asked respondents about the extent to which they desired and received "support and advice" regarding their "professional development and career" from others. We focused on the support that students reported receiving from other graduate students and from post-docs, the two groups with which they would be most likely to interact on a day-to-day basis. These indicators were combined into a three point scale with the highest value indicating that they received as much support as they wanted from both sources and the lowest indicating that they did not receive the desired level of support from either source. Student funding was measured by a single question that asked students to indicate the extent to which they believed that their financial support was "adequate to meet the cost of living where I live." Higher values on the five point scale indicated greater agreement. As detailed in the results section below, responses to individual items in each of the composite measures of graduate experiences were also examined as were responses to a question that asked about the percentage of financial support students received from a variety of sources.

Career commitment was measured through combining responses to two questions. The first asked students, “How likely are you to complete your current degree program?” with responses ranging on a five point scale from “definitely will not” to “definitely will.” The second asked, “Looking ahead, how likely is it that you will stay in the chemical sciences after you graduate?” with responses again ranging on a five point scale from “not at all likely” to “extremely likely.” Responses to these questions were skewed, with relatively few students indicating that they were unlikely to finish the degree or stay in the field. Thus, responses were combined into a three point scale with the highest point indicating that students definitely planned to finish the degree and remain in chemistry, the midpoint indicating that students definitely planned to finish but were only moderately likely to remain in the field, and the lowest value indicating that they did not believe they would finish the PhD.

Aspiration for a career as professor in a research-oriented university was measured by combining responses to questions regarding students’ career interests and plans to pursue a post-doctoral fellowship, which is a requirement for such a career goal. The data regarding career interests came from a question that began, “Please tell us about your current interest in the careers listed…” and was followed by a number of possibilities including “Professor (emphasis on research), Professor (emphasis on teaching), Researcher in industry, Researcher at government agency or national lab, Researcher (not professor) in college/university,” several administrative possibilities, teaching at the K-12 level and starting one’s own company. Respondents could indicate that they were “very interested, moderately interested, or not at all interested” in each possibility. The question regarding postdoctoral plans was phrased, “Do you plan to do a postdoctoral position upon completion of your Ph.D.?” with potential responses of yes, no and not sure. Responses to these questions were combined into a four-point scale with the highest value indicating they were very interested in both a post-doc and becoming a research professor and the lowest indicating no plans for either.

Independent and Control Variables. Dummy variables were used to measure status variables of gender, identification as an underrepresented minority, parental education, marital or partnered status, and having dependents. Parental education was used as an indicator of socio-economic status, and marital/partnered status and having dependents were used as

indicators of social support and responsibilities. Higher values on these variables (codes of 1 rather than 0) represented men, students who identified with a race-ethnic group other than Asian or non-Hispanic white, who reported having one or more parent completing college, were married or partnered, and who reported having "one or more dependent child (or adult) who received one half or more of their financial support" from them. Students' year in their graduate program was a continuous measure, ranging from 1 to 5.

Measures of students' career-related values, or preferences, were obtained from a series of questions that asked how important a variety of factors were in their "career planning process" and might influence their career plans. Three questions that involved areas related to remuneration and financial security ("finding a well-paying job," "having job security," and "finding a job that offers advancement opportunities") were highly correlated and combined into an additive scale (Cronbach's alpha = .65). Three other questions that involved areas related to family and other personal considerations ("having a job that gives me time for family, friends and hobbies," "desire to have a job in a certain geographical location," and "partner's professional circumstances") were also combined into an additive scale (alpha = .51).

While each of the individual-level variables described above was obtained from the ACS survey, the measures of departmental context came from other sources. Care was taken to obtain data from a time as close as possible to the date of the survey (2013). The amount of annual R and D expenditures and the number of postdoctoral and graduate students was obtained from the National Science Foundation's WebCASPAR database.[2] Data on the number of faculty were obtained from the Open Chemistry Collaborative in Diversity Equity (OXIDE).[3] The measures of size and funding were highly correlated, with larger departments more likely to receive substantial research funds. Thus the indicators were combined into an additive scale that represented departmental prestige and size, using standardized scores to adjust for different underlying metrics (Cronbach's alpha = .78). For descriptive results given in the text the departments were divided into three groups: those within the top quartile on this scale (38% of the students), those in the second quartile (32%), and those in the bottom half (30%).

[2] Data on the number of students were from 2016 and data on funding were from 2013. The data base is at https://ncsesdata.nsf.gov/webcaspar/

[3] Data were obtained at http://oxide.jhu.edu/2/demographics.

The OXIDE website also provided information on the number of faculty members who were women or identified as an underrepresented minority, resulting in two additional contextual measures: the percentage of faculty who were women and a dummy variable indicating whether or not a department had any faculty members who identified as URM. A dummy variable was used as a measure of URM presence on faculty instead of a percentage because the distribution of the percentage measure was very highly skewed. About one-fourth of the departments reported no faculty who identified as URM, and only 5 departments reported 10 percent or more. In a few cases departments did not respond to OXIDE's request for information, and a web search was conducted to find the missing data.

Finally, an indicator of each university's racial and ethnic diversity was obtained from an annual publication of U.S. News and World Report. It should be noted that this variable reflects the university in which a department was located rather than the chemistry department itself.[4]

As noted above, Cases were omitted if they had no data on the dependent measures or indicators of gender or identification as URM. With the independent variables missing values on continuous measures were recoded to the average (mean) value, and missing values on categorical variables were recoded to the modal category. This procedure was applied to one percent or fewer of the cases for each variable.

Analysis

We began the analysis by examining average values (means) of each of the independent variables by gender and identification as URM. Two way analyses of variance were used to determine both main and interaction effects. As noted in the text, testing for interaction effects was especially important to examine the possibility of what some theorists have called "intersectionality," the way in which the combination of different status characteristics, such as gender and identification as URM, might be related to opportunities and constraints (Collins, 2019). The computer program STATA was used for all analyses.

Mixed model regressions were used to examine the association of each dependent measure with gender and URM and the extent to which these associations were moderated by

[4] Data were obtained at https://www.usnews.com/best-colleges/rankings/national-universities/campus-ethnic-diversity.

the various control variables. Mixed models are especially appropriate for data sets in which elements of the sample (in this case students) are nested within other elements (departments). For each dependent measure (the three indicators of graduate school experiences and the two indicators of career plans) a series of models was examined: 1) a baseline, intercept only model; 2) a model that included only gender, identification as URM, and the interaction of these factors; 3) a model that added the other individual-level variables; 4) a reduced model that included only the individual-level independent variables that were statistically significant; 5) a model that added the measures of departmental context to this reduced model; 6) a model that added interactions of gender and identification as URM with measures of departmental context to model 5; and 7) a final reduced model that included only variables that were statistically significant and provided better fit than alternative models.

The intraclass correlation (ICC) at baseline and the variance associated with the constant term were used to quantify differences between departments. Model fit was determined by changes in the -2 log likelihood (-2 LL) statistic, which is distributed as chi-square, and a PRE statistic that quantifies changes in residual variance from one model to another. Because of their theoretical importance, indicators of gender and identification as URM were included in all models, even if they were not statistically significant. However, to preserve degrees of freedom, the interaction of these two variables was only included if it were statistically significant ($p<=.05$). Individual level variables that were significant in models that did not include the contextual variables were included in final models even if their association with the dependent measure fell below traditional thresholds of significance when additional measures were included. As a check on the findings, when results in Model 6 indicated significant interactions with a contextual variable, interactions that were not significant were removed to help determine the best fitting model and enhance degrees of freedom. Tables in the next section include model fit statistics for all models, but fixed effect coefficients for only the models that directly address the research questions. Fixed effects for intermediate models are available, upon request, from the corresponding author. The values for the adjusted means presented in figures in the text were obtained with the “margins” command within STATA.

Figure One illustrates the logic that underlay our analysis. We hypothesized that students' career plans were influenced by their graduate experiences and that both graduate experiences and career plans were influenced by students' individual characteristics and characteristics of the departments in which they studied.

Results

Table S-1 reports average values of the control variables for each of the four gender-URM sub-groups and the associated analysis of variance results. As noted in the text, students who identified as URM were significantly less likely to be in larger and more prestigious departments (t = 2.54, df = 1373, p (two-tail) = .01).

Analysis of Graduate Experiences

Table S-2 reports the average values on each measure of graduate experiences. Standard deviations for the total group are included, which were used to calculate the z-scores shown in Figure 1 and in the bottom panel of Table S-2. The multivariate analyses were, of course, conducted with the raw scores as the dependent measures. Z-scores were presented in the text only to have comparable metrics and enhance understanding of the results.

Table S-3 reports the model fit statistics for each measure of graduate experiences and the seven models described above. Table S-4 reports the fixed effect coefficients for the model with gender and URM (and, if appropriate the interaction) and the final, best fitting model (Model 7). The fixed effects associated with gender and URM in the two models can be compared to understand the extent to which differences related to gender and URM were moderated, or explained, by the control variables.

With the measure of advisor relations it can be seen that the fixed effect coefficients associated with gender and identification in the final best fitting model (Model 7) were very similar to those in Model 2, with a strongly significant interaction effect. Women, and especially those who identified as URM, were less likely to report supportive relationships with their advisors. In the baseline model a small, but statistically significant, proportion of the total variation in views of advisors was between departments (ICC=.03). Two contextual variables

were significantly related to advisor relations and, when these contextual variables were added to the model, the between-department variation was no longer significant.[5]

Table S-5 reports the average values for each individual item included in the composite measure of advisor relationships by gender and identification as URM and the analysis of variance results for each item. As noted in the text, these data illustrate the way in which less than optimal advisor-student relationships were reported by women, and especially URM women, across virtually all aspects of these relationships tapped within the survey.

Results related to the measure of interpersonal support are in Tables S-3 (second panel), S-4 (second set of columns) and S-6. Results of the mixed model analyses indicated that a model that included only gender and URM identification, as well as the interaction of these two variables, best fit the data. There was very little variation between departments (ICC=.004) and none of the individual level variables, nor any of the contextual variables, were significantly related to the amount of support students received from other students or post-docs. Thus, the coefficients shown in Table S-4 provide the best representation of factors associated with students' support. Data in Table S-6 provide descriptive information on the variable for each of the gender-URM groups. It shows the percentage of students who reported that they received the desired support from both peers and post-docs, from only one of those groups, or from neither group. The results illustrate the patterns behind the significant interaction effect with almost a quarter of the URM men reporting they received desired support from neither group.

Mixed model results regarding the adequacy of funding are summarized in the last panel of Table S-3 and the last set of columns in Table S-4, with additional analyses summarized in Tables S-7, S-8, and S-9. As explained in the text, the final, best fitting, model included an interaction of identification as URM with the scaled measure of departmental size and prestige, but substantial discrepancies related to URM identification remained in even the most prestigious schools (Figure 2 in the text). Table S-7 shows the percentage distribution of responses to the question regarding adequacy of funding by gender and identification as URM. Table S-8 reports the average percentage of financial support students received from teaching

[5] At baseline the random variance associated with the constant was significant at $z=2.08$, $p = .02$ (see top panel of Table S-3). But, as shown in Table S-4, in the final model this variance was no longer significant.

assistantships, research assistantships, fellowships, and personal resources such as loans, savings, other employment, and family members. Men were significantly more likely than women to have research assistantships; and non-URM men reported the largest percentage of support from this source (44.5% versus 39% for non-URM women, 40% for URM men and 35% for URM women). However, as noted in the manuscript, disparities in the need to rely on personal resources by URM status were much larger than those related to other types of support.

Table S-9 reports the results of additional analyses designed to check the extent to which different sources of funding might contribute to the greater reliance of URM students on personal resources. Model 1 reports results of a model including only URM status, Model 2 adds percentage of funds from TAs, RAs, and fellowships (omitting % from personal resources to avoid collinearity), and Model 3 adds dummy variables for departments ranked in the top 10 and from 11 to 50 (omitting those in the bottom 50). Model 4 includes only URM and the percentage of support from personal resources and Model 5 adds dummies for department rank to Model 4. In all models, the fixed effect associated with URM status remained highly significant. This indicates that, even when controlling for the source of funding, URM students were less likely to report having financial support that was adequate to meet their needs.

The discussion of the results related to funding notes substantial differences in net worth between members of racial-ethnic minorities that are underrepresented in STEM and others within the United States. Table S-14 summarizes the U.S Bureau of the Census data used to calculate the proportional difference. The first column of data gives the average net worth, based on all assets, within each race-ethnic group, and the second column gives data excluding home equity. The third and fourth columns compare the assets of minority households to non-Hispanic whites. The data for groups regarded as URM were combined, but it should be noted that the values for some sub-groups, particularly African Americans, are much lower.

While the individual and department-level control variables were not related to students' reports of support from others, several of these variables were related to students' reports of relationships with their advisors and adequacy of funding. (See the fixed effects associated with these variables in Table S-4.) Independent of gender and identification as URM,

students were more likely to report that they had positive relations with their advisors when they had been in their programs for fewer years, placed greater value on a career that was well-paid and secure, were in smaller and less prestigious departments, and in departments with a larger proportion of women faculty. Independent of gender and identification as URM, students less often reported adequate funding when they were married or partnered, had financial dependents, placed greater value on jobs that were secure and high paying, were in more diverse universities or in departments with at least one URM faculty member.

Career plans

Table S-10 reports the model fit statistics for the mixed model analyses of the measures of career plans, and Table S-11 reports the fixed effects for the models with only gender and URM as predictors and for the final best fitting model. Table S-12 reports the average values and associated standardized (z) scores of the dependent measures by gender and URM identification. Table S-13 reports the correlation coefficients (Pearson's r) between the independent and control variables in the analysis.

Inspection of the individual items involved in the measure of commitment indicated that, while two-thirds definitely planned to finish the PhD, only half of those (31% of the total group) said that they would definitely stay in the chemical sciences. Slightly more (36%) indicated that they either would not stay in chemistry or not finish the PhD. As explained in the text, men were significantly more likely than women to express high commitment, but, as can be seen by comparing the results of Model 2' and Model 7 in Table S-11 and as illustrated in Figure 3, these differences were moderated by relationships with advisors and departmental prestige. Baseline results also indicated that there were statistically significant differences between departments in students' commitment (ICC=.04, See Table S-10). These differences remained significant in the final model, but were partly moderated by the measure of departmental size and prestige and the interaction of this measure with gender. (See Table S-11 and the significant variance associated with the intercept.)

Slightly less than one-fifth of the students (19%) indicated that they were highly committed to pursuing a post-doc and very interested in becoming a professor with an emphasis on research. But substantially more (33%) reported that they did not plan to pursue a

postdoc or a career as a research professor. Over one-fourth of the men (26%) indicated that they were highly committed to pursuing a post-doc and very interested in becoming a professor with an emphasis on research, compared to only about a tenth (11%) of the women. The aspirations of men who identified as URM were especially notable, with average values on this variable that were over half a standard deviation greater than the overall mean (See Table S-12.) As explained in the text, and shown in Table S-11, gender differences in aspirations were only partially moderated by other variables. Differences related to identification as URM, however, were highly dependent upon the presence of URM faculty, as shown by the significant interaction effect and illustrated in Figure 4 in the text.

Several of the individual-level control variables were significantly associated with students' career plans, independent of gender and identification as URM. Students who were more advanced in their programs, married or partnered, placed greater value on a secure and well-paying job, and less value on a job that facilitated personal and family interests expressed greater commitment to finishing the degree and staying in the field. First-generation college students who placed less value on a job that supported family and other commitments were more likely to aspire to a professorship with an emphasis on research. Graduate experiences also were related to career aspirations, with having a supportive advisor enhancing both commitment to the field and aspirations to a tenure-track professorship. Interestingly, net of other variables, those who reported less personal support from others were more likely to aspire to a tenure-track professorship; and perceived adequacy of funding was not related to either commitment or aspirations. Finally, those who expressed greater commitment to finishing their degree and staying in the field were more likely, independent of other variables, to aspire to a professorship with an emphasis on research.

Future Research

This study focused on the way in which only two variables (gender and identification as URM) were associated with graduate experiences and career plans. Future research should examine other factors, such as gender identification, sexuality, and disability status. Further research should also examine experiences in Masters' degree programs, in non-ranked departments, and career plans beyond those associated with the professoriate, such as those

within industry or areas of the government. Focus on these additional areas is especially important given the tendency for URM and first-generation college students to more often be in Masters level programs and non-ranked departments.

Other aspects of the graduate school experience, from interactions within labs and other learning situations to policies related to areas such as funding, should be studied. Attention to nuances of interactions, such as those that incorporate or reflect implicit bias (1) and acknowledge the distinction of numerical diversity and true inclusion (2-3), could be especially informative. And, of course, it would be important to replicate this work in other academic areas, perhaps especially in those such as the physical sciences, where the representation of women and those who identify as URM is markedly less than in the population as a whole. In such replications, researchers should use sampling frames that result in adequate numbers of individuals within sub-groups. In the present study, some associations related to URM status were substantively strong but had relatively weak statistical significance, a result of the smaller number of students in that group.

Each of the four contextual variables in the analysis was related to at least one of the dependent measures, and the negative association of departmental size and prestige was especially notable. Future researchers should try to understand more about why these associations occurred. Do they reflect differences between more and less prestigious departments in terms of faculty actions, departmental culture, or policies? Results also suggest the important role of the diversity of faculties, and future research should try to understand why these associations appear. Do they simply reflect the aggregation of actions of individuals or might changing group compositions result in different departmental cultures and policies? How important is the role of "tokenism" within a department and is there a point at which a "critical mass" can help improve interactions (4-6)? Research on graduate experiences and plans in historically black colleges and universities (HBCUs) could be especially helpful in this area, as could research on the impact of an undergraduate education in an HBCU on graduate experiences and outcomes in STEM.

Future researchers should also examine a broader range of independent variables. No information was available on the characteristics of advisors, and it was not possible to test

hypotheses regarding the extent to which student-advisor affinity in research interests and status characteristics might impact relationships (7-8). The significant interaction effects between students' identification as URM and the presence of URM faculty in the analysis of students' aspirations could lend at least indirect support to this thesis. In addition, results indicated that students' family relationships and responsibilities, as well as their preferences regarding future careers, were associated with some graduate experiences and career plans, independent of gender, identification as URM, and departmental context. It is possible that these findings may reflect differences in felt obligations, life goals, and/or aspects of life development not tapped within this study. Understanding reasons for these associations could be a fruitful area of future exploration.

Perhaps most important is understanding how students who identified as URM maintained a greater commitment and higher aspirations than other students, especially in light of their significantly lower levels of funding and interpersonal support and, for women, poor relations with their advisors. What are the factors that help these students continue to pursue their goals? Does the process of coping with these factors within the scientific community differ from the process that is used within other areas of social life and interactions (9-10)? What are the costs of this process to individuals and to the discipline?

Finally, the results suggest that it is important to avoid simplistic generalizations about the associations of graduate school experiences and career aspirations with gender and identification as an URM. Clearly it would be inaccurate to assume, at least with this set of variables, that the types of inequities faced in graduate school related to URM status and gender are comparable from one variable to another or simply additive. For instance, women who identified as URM were least likely to report positive relationships with their advisors, but men who identified as URM were least likely to report receiving support from peers or adequate funding. Future research should devote attention to sorting out the associated complexities.

Implications for Policy and Practice

The results of our work have serious implications for changes in policies. We believe that concerted actions are needed to promote more equitable environments for all students

and to develop an inclusive and diverse STEM workforce that utilizes the full range of available talent.

Some issues could, conceivably, be quickly addressed. For instance, departmental administrators should be able to quickly identify and address issues involving discrepancies in student support. The chemistry discipline as a whole could develop suggestions for best practices and, conceivably, oversight mechanisms. Major funding agencies could develop policies to support more equitable funding outcomes.

Improving equity in other areas of the graduate experience will no doubt require more extensive efforts, but are, arguably, even more important. Departments could establish programs that promote more productive and supportive advisor-student relationships. Faculty could be taught how to be better advisors, perhaps motivated by the fact that their own reputations depend, in part, upon the success of their students. Students could be taught how to promote mentoring relationships and advocate for the help that they need. Department administrators could, and should, monitor the quality of these relationships and facilitate improvements. Disciplinary organizations, such as the ACS, could monitor the quality of help given to students, examine inequities, and promote change by recognizing individuals and departments that develop and sustain excellent advising.

One of the most disturbing finding of this analysis, and the one that should be of great concern to those involved in graduate education as well as all within the STEM community, was the extent to which students, and especially men, who identified as URM did not receive the support they desired from either graduate student peers or postdocs with whom they worked. In fact, none of the individual-level or contextual control variables were associated with this outcome. Many scientific breakthroughs are facilitated by interactions between colleagues and by sharing ideas, criticisms, and feedback. If these interactions involve only a portion of the available brainpower, the entire discipline loses. Such interactions also help temper the inevitable ups and downs that scientists experience within their work. When these benefits are limited to only some of the participants, all within that setting – students, post-docs, faculty, and staff – lose. In short, each relationship that is lost, or never begun, is a loss not just to individuals but to the discipline as a whole. In addition, the fact that some students must face,

on a recurring basis, the experience of exclusion and implicit devaluation, no doubt adds to other burdens that they face. For URM students, these burdens are extraordinarily high.

Yet, like other areas discussed in this section, these relationships can be changed. Such changes will no doubt require soul-searching and careful attention to the nature of day-to-day interactions and ways to minimize micro-aggressions, counter implicit biases, and promote truly positive and supportive professional relationships. They will require commitment to the creation of research groups and departments that are truly anti-racist and inclusive. As noted in the text, organizations within the scientific community, and elsewhere, are available to help in these endeavors.

Our findings related to career plans highlight the central role of student-advisor relationships and suggest that, with more supportive advising, gender differences in commitment to finishing the PhD degree and staying in the field may disappear. However, better advising was not sufficient to close the gender gap in aspirations. These differences remained substantively large and statistically significant even when students had highly supportive relationships with their advisors. The gap in aspirations was also independent of students' preferences regarding the extent to which future jobs would support personal and family-related activities. In other words, better advising or changing women's preferences regarding their jobs does not appear sufficient to deal with the gender discrepancies in aspirations. If chemistry departments and the field as a whole want to increase the representation of women in the professoriate to more closely match their representation among those who hold PhDs, it is important that they look at a range of factors that have been suggested as making the professoriate relatively less welcoming for women (11-12).

Finally, the associations with the various contextual measures suggest that it is important to confront the ways in which departmental cultures and norms reinforce inequities and, particularly, issues related to departmental prestige and faculty composition. Why are gender differences in career commitment exacerbated within the larger, more prestigious, and resource-rich departments? Why do differences in financial support related to students' identification as URM persist, albeit slightly muted, within these environments? These departments, which are viewed as leaders within the discipline, should have an obligation to

maintain the highest standards of equity. Perhaps the definition of prestige within the field should expand to include factors involved in the promotion of the long-term well-being of the discipline including, particularly, equitable treatment and support of students.

The impact of faculty diversity is also notable. As documented in the supplemental material, all students in departments with larger proportions of women faculty reported, on average, more positive relationships with their advisors. Having faculty who identified as URM enhanced the probability that students who identified as URM aspired to professorships with an emphasis on research. These findings suggest that, in addition to developing more equitable experiences for students, departments should continue to diversify their faculties. Ensuring more diverse faculties appears to enhance positive experiences for all.

Table S-1

Average Values of Measures of Status Characteristics, Values, and Contextual Variables by Gender and Identification as URM

	Women		Men		Total		F-Ratios		
Variables	URM	Non-URM	URM	Non-URM	Mean	SD	Gender	URM	Inter.
Status Variables									
Yrs in Prog.	2.70	2.50	2.48	2.56	2.54	1.17	0.47	0.33	1.62
Parent Ed.	0.65	0.76	0.58	0.71	0.72	0.45	2.38	8.11**	0.04
Married/Part.	0.43	0.40	0.31	0.44	0.42	0.49	0.76	1.15	2.74[a]
Dependents	0.13	0.04	0.11	0.11	0.08	0.27	1.22	2.82[a]	3.24[a]
Values									
Job	4.30	3.95	4.22	4.01	4.01	0.63	0.03	23.01***	1.41
Family and time	3.94	3.76	3.73	3.61	3.70	0.73	7.11**	4.65*	0.24
Context									
Size/Prestige	0.83	0.98	0.74	0.92	0.93	0.69	1.36	6.58**	0.04
Univ. Diversity	0.56	0.56	0.58	0.56	0.56	0.13	0.96	0.07	0.33
URM Fac	0.78	0.76	0.84	0.72	0.75	0.44	0.05	3.04[a]	2.00
Wom. Fac. (Prop)	0.19	0.19	0.18	0.18	0.18	0.06	2.6	0.01	0.06
N	63	590	64	658	1375				

***=p<.001, **=p<.01, *=p<.05, a=p<.10

Table S-2

Measures of Graduate School Experiences, Average Values and Z-Scores by Gender and Identification as URM

Average Values						
	Women		Men		Total	
	URM	Not URM	URM	Not URM	Mean	SD
Advisor	3.26	3.51	3.85	3.62	3.57	0.77
Support	1.40	1.46	1.17	1.50	1.46	0.82
Funding	3.35	3.85	3.20	3.80	3.77	1.11
Z-Scores						
	Women		Men			
	URM	Not URM	URM	Not URM		
Advisor	-0.39	-0.07	0.35	0.06		
Support	-0.07	0.00	-0.35	0.05		
Funding	-0.38	0.08	-0.51	0.03		

Table S-3

Model Fit Statistics, Measures of Graduate School Experiences Regressed on Status Characteristics, Values, and Contextual Variables

Advisor Helpful and Supportive			
	- 2 LL	Change	PRE
1) Baseline	3254.9		
2) Gender, URM, and Interact.	3231.8	23.18***	0.02
3) All Individual Level Vars.	3206.7	25.07***	0.02
4) RILM	3210.1	44.87***	0.03
5) RILM and CV	3196.6	13.49**	0.00
6) RILM, CV, and Interactions	3188.4	8.13	0.00
7) Final Reduced Model	3197.6	57.33***	0.03
ICC	0.03		

Support from Others at Desired Level			
Model	- 2 LL	Change	PRE
1) Baseline	2977.19		
2) Gender, URM, and Interact.	2964.72	12.47**	0.008
3) All Individual Level Vars.	2958.18	6.54	0.004
4) RILM	2964.72	12.47**	0.008
5) RILM and CV	2961.27	3.45	0.001
6) RILM, CV, and Interactions	2953.86	7.41	0.005
7) Final Reduced Model	2964.72	12.47**	0.008
ICC	0.004		

Funding Adequate			
	- 2 LL	Change	PRE
1) Baseline	4120.33		
2) Gender, URM, and Interact.	4098.71	21.62***	0.01
3) All Individual Level Vars.	4046.33	52.38***	0.03
4) RILM	4048.80	71.53***	0.04
5) RILM and CV	4031.05	17.75**	0.00
6) RILM, CV, and Interactions	4019.45	11.61	0.01
7) Final Reduced Model	4029.04	91.29***	0.05
ICC	0.14		

Note: Baseline model was intercept only model. Model 2 included gender, URM status, and the interaction of gender and URM. Model 3 added years in the program, parents' education, marital/partnered status, dependents, value placed on job security and salary, and value placed on family and time-related aspects of jobs. The reduced individual level models (RILM, Model 4) included variables that were significant in model 3. Model 5 added the contextual variables to the RILM, and Model 6 added interactions of the contextual variables and gender and URM status. If variables in the RILM were not significant when contextual level variables were added they were still included in the final reduced models (Model 7). The change in -2 LL and PRE statistic for Model 2, Model 4, and Model 7 were calculated by comparing to values for Model 1. For Model 3, the comparison was to Model 2, for Model 5 the comparison was to Model 4, and for Model 6 the comparison was to Model 5. a=p<.10 *=p<.05, **=p<.01, ***=p<.001.

Table S-4

Fixed and Random Effects, Mixed Model Regressions of Graduate School Experiences on Gender, URM Identification, and Control Variables

	Advisor Helpful				Support		Funding Adequate			
	Model 2		Model 7		Model 2		Model 2’		Model 7	
Indep. Vars.	b	s.e.	b	s.e.	b	s.e.	b	s.e.	b	s.e.
Male	.11*	0.04	.10*	0.04	0.03	0.04	-0.08	0.06	-0.04	0.06
URM	-.24*	0.10	-.28**	0.10	-0.07	0.09	-.45***	0.10	-.65***	0.15
Male#URM	.48***	0.15	.48***	0.14	-.26*	0.13	-----	-----	-----	-----
Individual-Level Controls										
Years Prog	-----	-----	-.06***	0.02	-----	-----	-----	-----	-----	-----
Mar/Part.	-----	-----	-----	-----	-----	-----	-----	-----	-.16**	0.06
Dependents	-----	-----	-----	-----	-----	-----	-----	-----	-.47***	0.11
Value Job	-----	-----	.10**	0.03	-----	-----	-----	-----	-.15***	0.05
Contextual Controls										
Dept. Prestige	-----	-----	-.10**	0.03	-----	-----	-----	-----	.13a	0.07
Sch. Diversity	-----	-----	-----	-----	-----	-----	-----	-----	-.87*	0.35
URM Faculty	-----	-----	-----	-----	-----	-----	-----	-----	-.28**	0.10
Women Fac.	-----	-----	.94*	0.41	-----	-----	-----	-----	-----	-----
Interactions										
URM*Prest	-----	-----	-----	-----	-----	-----	-----	-----	.31*	0.14
Intercept										
Constant	3.52***	0.04	3.18***	0.17	1.46***	0.03	3.78***	0.06	5.10***	0.28
Rand. Eff.			var.	s.e.	var.	s.e.	var.	s.e.	var.	s.e.
Constant	0.020*	0.009	0.009	0.007	0.001	0.004	.15***	0.04	.11***	0.03
Residual	.599***	0.024	.60***	0.02	.50***	0.02	1.08***	0.04	1.03***	0.04
ICC	0.03				0.004					
Model Fit Statistics										
Ch. -2 LL	23.18		34.15***		12.47***		21.45***		69.84***	
PRE	0.02		0.01		0.008		0.010		0.040	

Note: For model fit, model 1 was compared to baseline, and Model 2 to Model 1. Degrees of freedom equal the number of independent variables. *=p<.05, **=p<.01, ***=p<.001. Note that Model 2’ for the analysis of funding differs from Model 2 in Table S-3 by excluding the interaction of gender and URM, which was insignificant.

Supplemental Table S-5

Graduate Students Views of Advisors, by Gender and Identification as URM, Effect Sizes, and Analysis of Variance Results, Full Sample

Item	Not URM		URM		Total Group		ANOVA Results		
Fair and Supportive	Women	Men	Women	Men	Mean	SD	F-Gend/	F-URM	F-Inter.
Gives credit for contributions	4.09	4.12	3.63	4.27	4.08	1.09	7.08**	4.22*	5.65*
Advocates for me	3.72	3.78	3.51	4.02	3.75	1.18	4.26*	0.13	2.29
Supports career plan of choice	3.56	3.70	3.20	3.88	3.62	1.20	10.24***	1.36	4.02*
Models good professional relations	3.70	3.73	3.51	3.98	3.71	1.25	2.84a	0.02	2.14
Encourages me to take challenges	3.82	4.04	3.53	4.21	3.93	1.14	16.05***	0.56	3.83*
Encourages me to attain goals	3.83	3.96	3.49	4.18	3.89	1.17	11.91***	0.55	5.59*
Takes time to learn about me	3.13	3.14	2.75	3.52	3.13	1.36	6.24**	0.21	5.84*
Creates a fair environment	3.51	3.61	3.19	3.96	3.57	1.33	9.42**	0.08	5.15*
I get along well with my advisor	4.26	4.26	4.05	4.46	4.26	0.95	2.90a	0.39	2.79a
Satisfied with Student Work									
Advisor satisfied with my research	3.98	4.02	3.67	4.18	3.99	0.97	8.09**	0.92	5.77*
Advisor satisfied with my progress	4.06	4.07	3.70	4.27	4.06	0.93	10.38**	1	9.15**
Advisor sees me as top student	3.47	3.68	3.02	3.84	3.56	1.07	23.99***	2.78a	7.75**
Mentoring									
Helps develop professional relations	3.02	3.17	2.63	3.52	3.09	1.27	14.93***	0.4	6.69**
Provides info re. academic careers	2.84	2.98	2.54	3.38	2.91	1.26	11.85***	0.04	5.16*
Provides info re. non-acad. Careers	2.32	2.53	2.11	2.71	2.42	1.24	9.69**	0.23	1.77
Feedback Available and Given									
Gives regular feedback on research	3.80	3.88	3.60	4.02	3.84	1.16	4.89*	0.14	2.14
Regular feedback on degree progress	3.09	3.14	2.93	3.73	3.13	1.26	9.87**	1.77	7.11**
Satisfied with time have with advisor	3.76	3.81	3.37	4.05	3.78	1.26	10.14**	0.3	7.63**

Table S-5, Page 2

Item	Not URM		URM		Total Group		Analysis of Variance Results		
Engages in Academic Work	Women	Men	Women	Men	Mean	SD	F-Gender	F-URM	F-Inter.
Asks me to write 1st drafts	4.16	4.26	4.21	4.27	4.21	1.19	0.2	0.01	0.13
Engages me in writing proposals	2.56	2.87	2.72	3.00	2.73	1.41	3.23a	0.48	0.22
Encourages research presentations	3.63	3.86	3.47	4.04	3.75	1.26	10.33***	0	1.76
Understands Issues of Concern									
Advisor out of touch with career issues	2.66	2.74	2.70	2.61	2.70	1.32	0.01	0.16	0.55

Note: Responses given on a scale of 1 to 5 with higher values indicating greater satisfaction or agreement. Students with two advisors omitted, N=1,269.

Table S-6

Percent Distribution, Number of Peers and Post-Docs Providing Support Desired by Gender and Identification as URM

	Women		Men		
Category	URM	Non-URM	URM	Non-URM	Total
Neither Post-Docs nor Peers	16	12	23	13	13
Only One	29	30	36	24	28
Both Post-Docs and Peers	55	58	41	63	59
Total %	100	100	100	100	100
Total N	63	590	64	658	1375

Table S-7

Percentage Distribution, Adequacy of Funding, by Gender and Identification as URM

	Not URM			URM		
Response	Women	Men	Total	Women	Men	Total
Strongly Disagree	4	4	4	10	13	11
Disagree	12	11	12	24	23	24
Neither	6	13	10	8	11	9
Agree	50	44	47	40	38	39
Strongly Agree	27	28	27	19	16	17
Total %	100	100	100	100	100	100
Total N	590	658	1248	63	64	127

Table S-8

Average Percent of Funding by Source, Gender and URM Identification and Analysis of Variance Results

Average Percent				
Group	Teaching Assistantship	Research Assistantship	Fellowships	Personal Resources
URM Women	33.0	34.5	25.4	7.0
Non-URM Women	33.0	38.6	23.4	3.2
URM Men	28.0	39.7	24.0	7.1
Non-URM Men	30.8	44.5	18.7	3.2
Total	31.7	41.3	21.3	3.5
Analysis of Variance Results				
Source of Variation	Teaching Assistantship	Research Assistantship	Fellowships	Personal Resources
F-Gender	1.94	3.75*	1.22	0.00
F-URM	0.28	2.33	1.79	14.89***
F-Interaction	0.28	0.02	0.34	0.00

Table S-9

Mixed Model Regressions of Funding Adequacy on URM, Source of Funding, and School Rank

	Model 1		Model 2		Model 3	
Ind. Var.	B	s.e.	b	s.e.	b	s.e.
URM	-.45***	0.1	-.44***	0.1	-.44***	0.1
TA	-----	-----	.009***	0.002	0.009***	0.002
RA	-----	-----	.012***	0.002	0.012***	0.002
Fellow	-----	-----	.014***	0.002	0.014***	0.002
top 10			-----	-----	0.11	0.16
11 to 50			-----	-----	0.11	0.1
Constant	3.74***	0.05	2.66	0.16	2.59***	0.17
var const	.15***	0.04	.134***	0.035	0.13	0.03
var resid	1.08***	0.04	1.024	0.041	1.02	0.04
-2 LL	4100.71		4028.19		4026.9006	
Change in -2 LL	19.62		72.52			
PRE	0.007		0.052		0.004	

	Model 4		Model 5	
Ind. Var.	b	s.e.	b	s.e.
URM	-.36***	0.1	-.36***	0.1
Personal Resources	-.03***	0.003	-.03***	0.003
top 10	-----	-----	0.13	0.15
11 to 50	-----	-----	0.10	0.1
Constant	3.84***	0.05	3.77***	0.08
var const	0.12***	0.03	.12***	0.03
var resid	1.01***	0.04	1.01***	0.04
-2 LL	4006.35		4005.06	
Change in -2 LL	94.36		1.29	
PRE	0.064		0.000	

Note: Models 1 and 4 are compared to baseline. At baseline, intercept only, -2 LL = 4120.33, variance of constant = .166, s.e. = .0415; variance of residual = 1.088, s.e. = .043. Model 3 compared to Model 2 and Model 5 compared to Model 4.

Table S-10

Model Fit Statistics, Measures of Career Commitment and Aspirations Regressed on Status Characteristics, Values, and Contextual Variables

Commitment to Finishing Degree and Chemistry			
Model	- 2 LL	Change	PRE
1) Baseline	3348.0		
2) Gender, URM, and Interact.	3333.5	14.45**	0.01
3) All Individual Level Vars.	3110.3	223.23***	0.15
4) RILM	3118.2	229.74***	0.15
5) RILM and CV	3112.9	5.38	0.00
6) RILM, CV, and Interactions	3107.3	5.54	0.00
7) Final Reduced Model	3111.0	236.99***	0.15
ICC	0.04		

Aspirations to Research Professorship			
Model	- 2 LL	Change	PRE
1) Baseline	4173.75		
2) Gender, URM, and Interact.	4091.53	82.22***	0.06
3) All Individual Level Vars.	3888.25	203.28***	0.13
4) RILM	3897.30	276.45***	0.17
5) RILM and CV	3891.99	5.32	0.00
6) RILM, CV, and Interactions	3883.69	8.30	0.01
7) Final Reduced Model	3890.70	283.05***	0.18
ICC	0.02		

Note: Baseline model was intercept only model. Model 2 included gender, URM status, interaction of gender and URM, years in the program, parents' education, marital/partnered status, dependents, value placed on job security and salary, value placed on family and time-related aspects of jobs, and the measures of graduate school experience. For analysis of aspirations to a research professorship the measures of commitment and confidence were also added to models. The reduced individual level models (RILM) included variables that were significant in model 2. Model 4 added the contextual variables to the RILM, and Model 5 added interactions of the contextual variables and gender and URM status. If variables in the RILM were not significant when contextual level variables were added they were still included in the final reduced models (Model 6). The change in -2 LL and PRE statistic were calculated by comparing to values for Model 1. For Model 4 these statistics were calculated with comparisons to Model 3, and for Model 5, the comparison was model 4. *=p<.05, **=p<.01, ***=p<.001.

Table S-11

Fixed Effects from Mixed Model Regressions of Measures of Career-Related Plans on Gender, Identification as URM, Graduate Experiences, and Control Variables

	Commitment				Aspire Research Prof.			
	Model 2’		Model 7		Model 2’		Model 7	
Indep. Vars.	b	s.e.	b	s.e.	b	s.e.	b	s.e.
Male	.15***	0.04	-0.02	0.07	.51***	0.06	.39***	0.05
URM	0.12	0.08	0.09	0.07	.21*	0.10	-0.23	0.21
Individual-Level Controls								
Years Prog	-----	-----	.06***	0.02	-----	-----	-----	-----
Parents' Ed	-----	-----	-----	-----	-----	-----	-.19***	0.06
Mar./Part.	-----	-----	.18***	0.04	-----	-----	-----	-----
Value Job	-----	-----	.16***	0.03	-----	-----	-----	-----
Value Fam	-----	-----	-.06*	0.03	-----	-----	-.13***	0.04
Graduate Experiences and Commitment								
Advisor	-----	-----	.33***	0.03	-----	-----	.18***	0.04
Support	-----	-----	-----	-----	-----	-----	-.08*	0.04
Commitment	-----	-----	-----	-----	-----	-----	.36***	0.03
Contextual Controls								
Dept. Prest.	-----	-----	-.10*	0.05	-----	-----	-----	-----
URM Fac.	-----	-----	-.10[a]	0.06	-----	-----	-.14*	0.07
Interactions								
Male*Pres.	-----	-----	.10[a]	0.06	-----	-----	-----	-----
URM*Fac.	-----	-----	-----	-----	-----	-----	.48*	0.24
Intercept								
Constant	1.86***	0.04	0.2	0.19	1.97***	0.05	1.51***	0.20
Random Eff.	var.	s.e.	var.	s.e.	var.	s.e.	var.	s.e.
Intercept	0.023**	0.011	0.015*	0.008	0.018	0.015	0.002	0.01
Residual	0.644***	0.025	.551***	0.022	1.134***	0.045	.99***	0.04
ICC	0.035				0.015			
Model Fit Statistics				-				
Ch. in -2 LL	14.33***		222.66***		79.95***		203.11***	
PRE	0.01		0.14		0.06		0.13	

Note: Model 1 compared to baseline, model 2’ to Model 1. a=p<.10, *=p<.05, **=p<.01, ***=p<.001. Note that Model 2’ differs from Model 2 in Table S-10, because Model 2’ omits the interaction of gender and URM identification. This interaction was not significant with the analysis for either dependent variable in this model.

Table S-12

Measures of Career Commitment, Average Values by Gender and Identification as URM and Associated Z-Scores

	Average Values					
	Women		Men		Total	
	URM	Non-URM	URM	Non-URM	Mean	SD
Commitment	1.95	1.86	2.16	2.01	1.95	0.82
Aspirations	2.05	1.98	2.83	2.47	2.26	1.10

	Z- Scores				
	Women		Men		-
	URM	Non-URM	URM	Non-URM	
Commitment	0.00	-0.11	0.25	0.07	
Aspirations	-0.19	-0.25	0.52	0.19	

Supplemental Table S-13

Pairwise Correlations of Variables Included in Models

Variables		1	2	3	4	5	6	7	8	9	10	11	12	13	14	15	16	17
1)	Male	1.00																
2)	URM	0.01	1.00															
3)	Advisor	.09***	-0.02	1.00														
4)	Support	0.01	-.08**	.08*	1.00													
5)	Funding	-0.03	-.15***	.14***	.11***	1.00												
6)	Confident	.20***	.10***	.29***	.09***	-0.01	1.00											
7)	Committed	.09***	0.04	.32***	.06*	0.04	.31***	1.00										
8)	Aspirations	.23***	0.05	.23***	-0.03	0.01	.20***	.34***	1.00									
9)	Yrs. Prog.	0.01	0.01	-0.09***	0.02	-.07*	0.05	.08**	0.04	1.00								
10)	Par. Ed	-.06*	-.07**	-0.02	-0.03	0.04	-.07*	-.06*	-.10***	-0.01	1.00							
11)	Mar/Part	0.03	-0.01	0.01	0.01	-.11***	.11***	.13***	.06*	.12***	-0.05	1.00						
12)	Dependents	.12***	0.05	0.03	-0.02	-.15***	.10***	.11***	.05*	.14***	-.09***	.31***	1.00					
13)	Val. Sec. Job	0.04	.12***	0.09***	-0.04	-.13***	.18***	.16***	0.03	-0.01	-.11***	0.03	.06*	1.00				
14)	Val Fam./Pers.	-.10***	0.07	0.01	0.01	-0.02	-0.02	-0.02	-.10***	0.00	-0.01	.10***	-.06*	.16***	1.00			
15)	Dept. Prestige	-0.05	-.07**	-.08**	0.03	.11***	-.12***	-.09**	-0.04	-0.01	.18***	-.08**	-.12***	-.13***	0.00	1.00		
16)	Diverse Univ.	0.02	0.01	-.06*	-0.01	-.06*	0.01	-0.04	0.01	0.01	.13***	-0.05	-.07*	-.07*	0.02	.25***	1.00	
17)	Prop. Wom. Fac	-.06*	0.01	.08**	0.01	-.07**	0.00	0.03	0.01	-0.03	-0.03	0.00	-0.02	0.04	0.01	-0.02	-.23***	1.00
18)	Any URM Fac	-0.04	0.04	0.03	-0.04	-.10***	0.00	-0.04	-0.05	0.00	0.00	0.01	0.01	0.02	0.05	0.04	0.02	.32***

Note: N=1388, *=p<.05, **=p<.01, ***=p<.001.

Table S-14

Net Worth by Race and Hispanic Origin, 2017

Race and Hispanic Origin of Householder	Average Net Worth: All Assets	Average Net Worth: Excluding Home Equity	% of White, Non-Hispanic: All Assets	% of White, Non-Hispanic: Excluding Home Equity	Number of Housholds (1000's)
White alone, not Hispanic	171,700	70,240	-----	-----	100,200
Black alone	9,567	2,827	0.06	0.04	84,440
Asian alone	157,400	61,410	0.92	0.87	17,030
Other (residual)	37,660	8,733	0.22	0.12	6,463
Hispanic origin (any race)	25,000	7,108	0.15	0.10	4,040
URM (Black, Other, Hispanic)	19,556	5,402	0.11	0.08	38,880

Data obtained from Tables 1 and 4, https://www.census.gov/data/tables/2017/demo/wealth/wealth-asset-ownership.html. URM category includes Blacks, Others, and Hispanic origin

Figure 1: Model That Guided the Analysis of Chemistry Graduate Students' Perceptions of their Graduate School Experiences and Career Plans

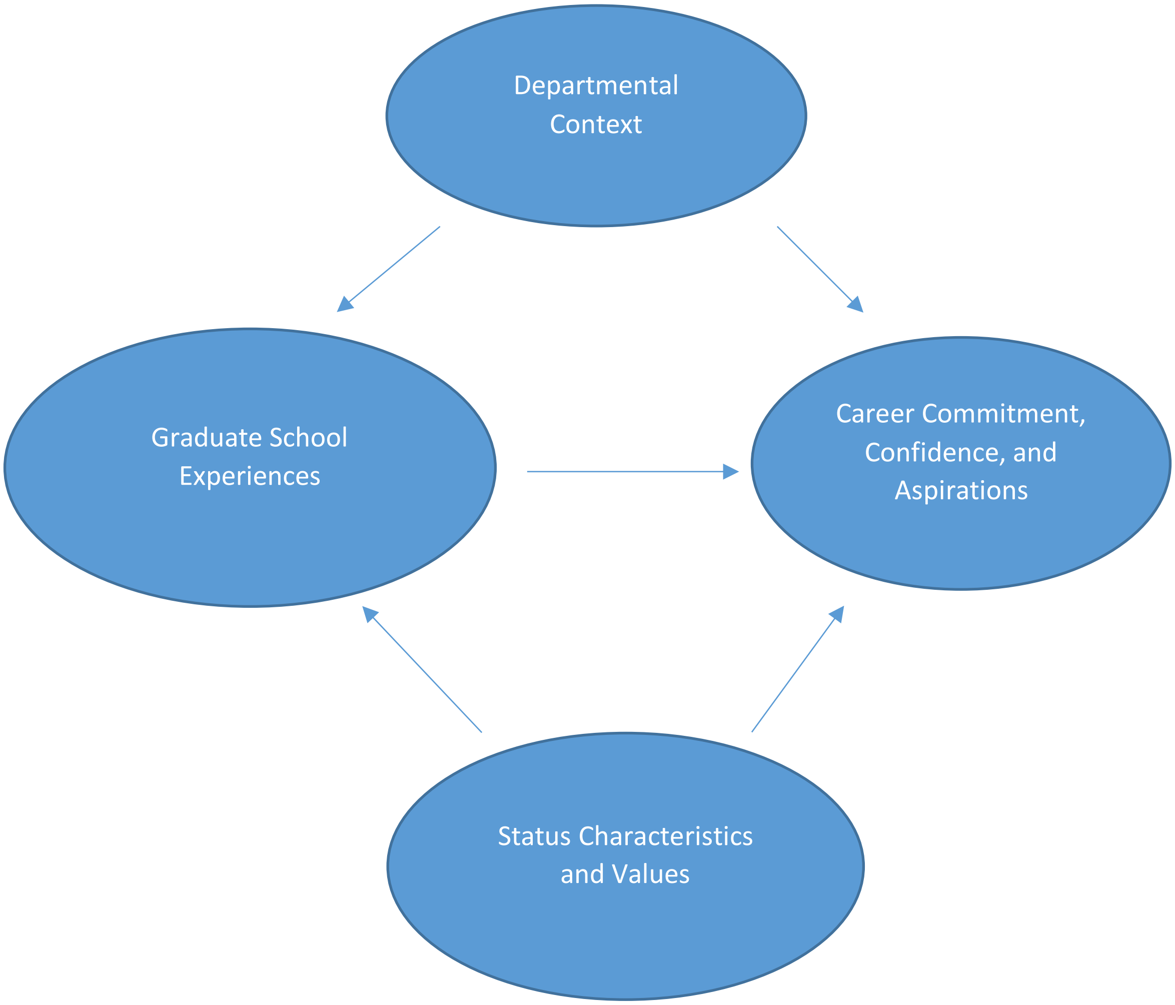

References

1. Moss-Racusin, Corinne A., Dovidio, John F., Victoria L. Brescoll, Mark J. Graham, and Jo Handelsman. 2012. Science Faculty's Subtle Gender Biases Favor Male Students. *Proceedings of the National Academies of Sciences*. 109 (41): 16474-16497.

2. Purrity, Chandler, Lynette R. Strickland, Eanas Alla, Benjamin Blonder, Emily Klein, Michael T. Kohl, Earyn McGee, Maclovia Quintana, Robyn E. Ridley, Beth Tellman, and Leah R. Gerber 2019. "Without Inclusion, Diversity Initiatives May Not Be Enough." *Science* 357(6356): 1101-1102.

3. Roberson, Quinetta M. 2006. "Disentangling the Meanings of Diversity and Inclusion in Organizations." *Group and Organization Management. 31*(2): 212-236.

4. Budig, Michelle J. 2002. "Male Advantage and the Gender Composition of Jobs: Who Rides the Glass Escalator?" *Social Problems 49*(2): 258-277.

5. Kanter, Rosabeth Moss. 1977. *Men and Women of the Corporation*. New York: Basic Books.

6. Spangler, Eve, Marsha A. Gordon, and Ronald M. Pipkin. 1978. "Token Women: An Empirical Test of Kanter's Hypothesis." *American Journal of Sociology 84*(1): 160-170.

7. Carrell, Scott E., Marianne E. Page, and James E. West. 2010. "Sex and Science: How Professor Gender Perpetuates the Gender Gap." *The Quarterly Journal of Economics 125*(3): 1,101-1,144.

8. Mouw, Colleen B., Sarah Clem, Sonya Legg, and Jean Stockard. 2018. "Meeting Mentoring Needs in Physical Oceanography: An Evaluation of the Impact of MPOWIR. *Oceanography* 31(4): 105-113.

9. Brown, Danice L. and Tracy L. Tylka. 2011. "Racial Discrimination and Resilience in African American Young Adults: Examining Racial Socialization as a Moderator. *Journal of Black Psychology* 37(3):259-285.

10. Shavers, Marjorie C. and James L. Moore III. 2014. "Coping and Resiliency Strategies of African American Women Enrolled in Doctoral Programs at Predominately White Institutions. *Frontiers: A Journal of Women Studies* 35(3): 15-38.

11. Greene, Jessica, Priscilla Lewis, Geraldine L. Richmond, and Jean Stockard. 2010. "Is the Academic Climate Chilly? The Views of Women Academic Chemists" *Journal of Chemical Education*, 87(April): 381-385.

12. Stockard, Jean, Jessica Greene, Geraldine Richmond, and Priscilla Lewis. 2018. "Is the Gender Climate in Chemistry Still Chilly? Changes in the Last Decade and the Long-Term Impact of COACh Sponsored Workshops," *Journal of Chemical Education* 95 (9): 1492-1499.
